# Infertile women's experience of stressful dilemmas during assisted reproductive technology treatment

**DOI:** 10.3389/frph.2026.1886332

**Published:** 2026-08-20

**Authors:** Li Deng, Ya Li, Fang Wang, Jitao Zeng, Yuyan Li, Wei He, Jiarui Yao, Shan Meng

**Affiliations:** Reproductive Medical Center, The First Affiliated Hospital (Southwest Hospital) of Army Medical University, Chongqing, China

**Keywords:** assisted reproduction, infertility, qualitative research, stress dilemma, women's mental health

## Abstract

**Objective:**

This study aimed to explore the unique stress experiences and multidimensional stress dilemmas of infertile women during assisted reproductive technology (ART) treatment in the Chinese cultural context.

**Methods:**

Fifteen infertile women undergoing ART treatment at a tertiary hospital in Chongqing were recruited for semi-structured face-to-face interviews. Guided by descriptive phenomenology, interview data were analyzed using the Colaizzi phenomenological approach with NVivo software.

**Results:**

Three core stress dimensions were identified; while these stress categories align with findings from Western research, their experiential manifestations are shaped by local Chinese sociocultural contexts: subjective stress dilemmas (negative emotions, body image distress, cognitive biases, and internalized stigma shaped by traditional fertility norms), objective stress dilemmas (work–treatment conflicts, limited family/spousal support, and heavy financial/time burdens under local social policies), and coping dilemmas (ineffective self-regulation and insufficient culturally tailored external support).

**Conclusion:**

Stress-related struggles reported by sampled Chongqing women share common stress categories documented in Western cohorts, yet their concrete manifestations and perceived severity are distinctly shaped by local Chinese sociocultural norms. These findings highlight the necessity of developing culturally sensitive psychological interventions and social support strategies for infertile women in China, providing a novel localized perspective for global reproductive mental health research.

## 1 Introduction

With advances in medical technology, assisted reproductive technology (ART) has enabled many infertile families to achieve pregnancy, establishing itself as a mainstream therapeutic option. With the adjustment and optimization of China's fertility policies, the two-child policy has been fully liberalized, and the three-child policy was officially implemented in 2021 (1). From the perspective of traditional Chinese culture, having children has always been a core issue in family life. In traditional Chinese culture, there is the traditional concept that “having no offspring is the gravest of the three unfilial acts”, a concept rooted in the ancient social and cultural backgrounds and family values (2). In the social system of that era, the continuation of the family line was regarded as of vital importance. Despite profound changes in family structures and values in modern Chinese society, procreation remains a central element of family life (3). Against this cultural backdrop, women with infertility face multiple sources of pressure from their families. The strong desire and expectation for offspring from family elders, as well as the subtle changes and new conflicts that may emerge in marital relationships due to fertility issues, are major sources of psychological stress for these women (4). These culture-derived stressors can exert a profound impact on the psychological state and treatment experience of women undergoing ART. The operational definition of stress dilemma in the present study is modified from predicament theory in health sociology (5), which conceptualizes dilemma as a situational conflict between two unfavourable alternatives, distinct from singular stressors that merely create passive strain rather than conflicting choices.

In the international research field, studies on the psychological impact of assisted reproductive technology on infertile women have yielded relatively rich results. Numerous international scholars have conducted in-depth explorations of the psychological status of infertile women during ART treatment from an interdisciplinary perspective integrating psychology, sociology and other disciplines. In terms of psychological research, existing studies have quantified the impacts of assisted reproduction treatment on infertile women's psychology through scientific psychological assessment tools and rigorous research methods, and found that infertile women are highly susceptible to mood disorders, such as anxiety and depression, during the process of assisted reproduction treatment (6). In sociological research, scholars have also noted the significant impact of social support systems on the psychological state of infertile women during ART treatment, and found that adequate social support can effectively alleviate their psychological stress and help them better cope with the challenges of treatment (7, 8). However, despite notable progress in theoretical construction and empirical research in international studies, it is important to note that significant differences exist between China and other countries in terms of cultural backgrounds, social attitudes, and healthcare systems. For example, in Western societies, cultural attitudes are more tolerant of individual reproductive choices, and societal perceptions of infertility differ significantly from those in China (9). These differences mean that the findings of international studies cannot be directly applied to Chinese infertile women, and thus need to be re-examined and reinterpreted in the context of China's local cultural and social realities.

In summary, conducting in-depth research on the stress dilemmas of Chinese infertile women undergoing ART treatment is of great practical and academic significance. At the practical level, an in-depth analysis of the psychological stress of infertile women during ART treatment can provide a scientific basis for improving ART-related medical services and a reference for clinicians to develop targeted psychological support strategies in clinical practice. This can further optimize the treatment experience of infertile women and improve the overall success rate of ART.

From an academic perspective, this research can enrich localized research findings in China's ART field, establish a research system with Chinese characteristics, provide unique perspectives and case studies for cross-cultural research, facilitate global communication and integration in this research field, and promote the further development and in-depth exploration of related research.

Based on the above, this study adopted a qualitative research approach, adhered to a rigorous academic attitude, and explored ART-related stress experiences among Chongqing sampled women, focusing on how indigenous Chinese culture shapes the manifestations of common infertility-related stressors widely reported in Western literature. The aim was to comprehensively and realistically portray the psychological status of this special group, thereby laying a solid theoretical foundation for subsequent research and practical applications.

## 2 Method

### 2.1 Participants

Purposive sampling was used to recruit infertile women receiving ART treatment at the reproductive medicine center of a tertiary hospital in Chongqing, China, for face-to-face interviews. The sample size was determined based on the principle of data saturation.

Inclusion criteria: (1) age 18–45 years old; (2) Women with clinically diagnosed infertility who were undergoing ART treatment, Participants were recruited across distinct treatment phases including ovarian stimulation, oocyte retrieval and post-embryo transfer phases; some participants also completed multiple subsequent frozen embryo transfer cycles. Participants were enrolled at different treatment phases to comprehensively capture physical discomfort, body image disturbance and emotional fluctuations emerging throughout the full ART pathway. (3) They could fully participate in interviews and had intact cognitive, reading and comprehension abilities, comprehension, thinking and judgment abilities. Exclusion criteria: (1) History of diagnosed moderate-to-severe mental disorders or severe cognitive dysfunction confirmed via previous psychiatric medical records. Per the institutional reproductive center clinical management specification of the study hospital, patients with uncontrolled severe mental disorders were excluded for ART implementation due to unstable mental status that cannot guarantee informed treatment decision-making and treatment compliance; those with mild, well-controlled mental disorders under stable medication follow-up were not excluded from the present study. (2) Severe impairment of vital organ function

The participants presented obvious heterogeneity in age, educational background, marital status and number of ART cycles. Data saturation was assessed progressively during sequential interviews: no new core themes or subcategories emerged from the 12th interview onwards. Three further interviews (13th to 15th) were conducted for verification, which only yielded repeated content and did not generate new experiential themes. Thus, a total of 15 participants were finally enrolled. The demographic characteristics of the study participants are summarized in Table 1. This study was approved by the Institutional Review Board (IRB) of the hospital (approval number: BIIT2025270KX). All participants provided written informed consent and volunteered to participate in this study.

**Table 1:** Characteristics of semi-depth interview participants.

Number	Age	Educational attainment	Occupation	Marital Status	Pregnancy and Delivery History	Number of cycles	Infertility factors
N1	37	Junior college	Unemployed	First marriage	G_3_P_1_	7	Female factors
N2	35	Undergraduate	Unemployed	First marriage	G_1_P_1_	3	Female factors
N3	36	Undergraduate	Police	First marriage	G_1_P_1_	4	Female factors
N4	34	Undergraduate	Unemployed	First marriage	G_1_P_1_	4	Female factors
N5	30	Undergraduate	Unemployed	Remarriage	G_5_P_0_	3	Female factors
N6	33	Junior college	Employee	Remarriage	G_2_P_0_	3	Both factors
N7	28	Junior High School	Unemployed	Remarriage	G_0_P_0_	3	Female factors
N8	30	Junior college	Unemployed	First marriage	G_0_P_0_	2	Male factors
N9	29	Junior college	Unemployed	First marriage	G_0_P_0_	8	Female factors
N10	32	Junior college	Civil servant	First marriage	G_2_P_1_	3	Female factors
N11	38	Junior High School	Unemployed	Remarriage	G_3_P_0_	2	Female factors
N12	32	Master's degree	Civil servant	First marriage	G_0_P_0_	2	Female factors
N13	32	Undergraduate	Accounting	First marriage	G_1_P_0_	4	Female factors
N14	32	Junior college	Unemployed	First marriage	G_2_P_0_	2	Female factors
N15	36	Junior High School	Salesperson	First marriage	G_2_P_2_	2	Female factors

### 2.2 Develop the interview guide

A preliminary interview guide was developed based on the research objectives through a comprehensive literature review and group discussions among the research team. Prior to the formal interviews, two pilot interviews were conducted with infertile women who met the inclusion criteria. Based on the pilot interview findings, the formal interview guide was revised and finalized, with the specific content presented in Table 2.

**Table 2:** Interview guide.

Serial Number	Problem
1	Can you tell me about what led you to pursue fertility treatment?
2	What prompted you to start considering assisted reproductive technology?
3	How supportive has your spouse been throughout the IVF process? Are your family and friends aware of the situation? How has their attitude affected you?
4	Do you feel pressured by societal expectations? Have you chosen to conceal your treatment? What are the reasons?
5	How has physical discomfort during treatment affected your daily life and emotional well-being?
6	What situations most frequently trigger intense emotional fluctuations? How do you handle them?
7	Has the treatment altered your perception of your self-identity?

### 2.3 Procedure

Semi-structured face-to-face interviews were used for data collection in this study, and all interviews were conducted by the same researcher to ensure consistency in the research process. Interviews were conducted in a private, quiet room (e.g., an unused conference room) at the hospital. Before each interview, the researcher explained the study's purpose and methodology to the participants, obtained their verbal consent, and then activated the recording device after the participants signed the written informed consent form in person.

During each interview, the researcher adopted non-participant observation without intervening in participants' narration; the researcher not only paid close attention to the participants' responses, but also closely observed and documented their facial expressions, body language, emotional states and other non-verbal behaviors throughout the whole interview process. All nonverbal observational information was recorded in written notes immediately alongside audio transcription after interviews. The researcher adhered to the principle of value neutrality and avoided subjective judgments. Meanwhile, the researcher flexibly probed further into the topics based on the participants' narratives, encouraged them to express their true thoughts in depth, and adjusted the order of interview questions to obtain more in-depth information.

Each interview ranged from 15 to 40 min, with a median duration of 29 min and modal length of 32 min. Only two interviews were completed in less than 20 min; these two participants had concise expression habits and quickly exhausted all personal relevant experience after core questions. Despite several interviews shorter than 20 min, the total dataset achieved full data saturation consistent with Colaizzi phenomenology requirements: all core experiential dimensions were repeatedly mentioned across participants, no new themes emerged upon extra supplementary interviews. Additionally, pre-set semi-structured questions were highly targeted focusing on lived stress experience; combined with real-time flexible probing and field non-participant observation records, short-duration interviews still collected complete first-person experiential information sufficient for extracting essential structural features required by descriptive phenomenological analysis.

### 2.4 Data analysis

Within 24 h of each interview, the researcher repeatedly listened to the audio recordings, transcribed them verbatim into textual data, and simultaneously documented non-verbal information (e.g., changes in participants' facial expressions and body language) observed during the interview. All researchers were clinical staff at the participating reproductive medicine center, and interviews were conducted by a single in-house clinician with no pre-existing therapeutic ties to participants. Participants were fully informed of voluntary enrolment and guaranteed unchanged routine clinical care to reduce response bias stemming from researcher–patient affiliation. To offset interpretative bias derived from the team's clinical expertise, neutrality was highlighted at each interview outset, and inter-coder cross-verification involving non-clinical team members was adopted during thematic analysis. Following phenomenological epoche requirements, all investigators documented prior experience-based preconceptions before coding; these pre-established assumptions were archived independently and periodically reviewed throughout analysis to curb subjective interpretation.

This study adopts descriptive phenomenology as the overarching philosophical framework. This philosophical orientation aims to objectively present participants' authentic lived experiences and the essential structures of their stress dilemmas, which is fully consistent with the traditional descriptive nature of the Colaizzi seven-step analytical approach (10). The analysis process is as follows: (1) Read the interview transcripts carefully and take detailed reflective notes; (2) Screen and extract meaningful statements that align with the research phenomenon; (3) Summarize and extract the core meaning from the meaningful statements; (4) Identify common conceptual themes and semantic features to develop core themes, sub-themes and analytical categories; (5) Link the identified themes to the research phenomenon and provide a comprehensive descriptive account; (6) Illustrate the essential structural characteristics of the research phenomenon; (7) Return to the original interview data to verify the authenticity and credibility of the themes. Several rigorous strategies were adopted to guarantee the trustworthiness of qualitative data analysis. Specifically, member checking was performed by feeding preliminary thematic outcomes back to five randomly chosen participants to validate the consistency between analytical conclusions and their authentic treatment experiences; peer debriefing was implemented whereby three research members coded interview transcripts independently, with divergent coding outcomes reconciled through group discussion.

Negative case analysis was also conducted throughout the analytical process: we deliberately screened and examined individual narratives that contradicted the preliminary themes. Two participants held relatively mild stress perceptions and rarely reported work or financial burdens, which differed from the dominant experiences of the sample. These divergent cases were not excluded; instead, they were fully re-analyzed and incorporated to refine the final thematic framework, ensuring the findings reflect the full spectrum of participants' experiences. Meanwhile, a complete audit trail was established via systematic archiving of raw interview recordings, verbatim transcripts, coding manuscripts and field observational notes to preserve traceable documents for future inspection.

## 3 Results

Fifteen participants were recruited for face-to-face interviews using purposive sampling, as described in the Methods section. Half of the participants were aged 28–37 years, and approximately 50% of them had infertility attributed to female factors. Detailed demographic and clinical characteristics of the participants are summarized in Table 1.

### 3.1 Theme 1: subjective stress dilemma

#### 3.1.1 Negative emotional distress

ART treatment is characterized by long cycles and uncertain outcomes, which meant participants commonly experienced significant mood swings and negative emotions (e.g., anxiety and worry) during treatment. Such negative emotions were particularly pronounced during the critical stages of oocyte retrieval and post-embryo transfer. *N13: “I initially thought I would definitely succeed once I started treatment, without considering the possibility of failure. When oocyte fertilization failed, I experienced intense anxiety.” N12: “Having 28 oocytes retrieved in a single cycle but still failing to conceive left me feeling immense grief and distress.” N11: “I was anxious about the limited number of available embryos and feared repeated treatment failures; both of which intensified my anxiety about future treatment attempts.”*

#### 3.1.2 Body image changes dilemma

Most participating women experienced negative changes in body image during ART treatment, which caused significant psychological distress. The main manifestations included weight gain, induration at injection sites, and concerns about the potential carcinogenic risks of ART medications.

*N11: “I gained a lot of weight during treatment and haven't been able to lose it despite trying various methods. This change in my body image has made me lose my self-confidence.” N5: “I was more worried about hard lumps forming in specific areas of my body from long-term injections than about the injection pain itself, which made me extremely anxious about my physical health.” N4: “Frequent blood tests led to bruising because of my fragile blood vessels, which was not only physically uncomfortable but also distressing to look at.” N3：“Things I read online about ART drugs causing cancer and weight gain left me in a constant state of fear during treatment and made me extremely worried about my health.”*

#### 3.1.3 The cognitive bias dilemma

Many participating women had limited and inaccurate knowledge of ART prior to treatment, with most of their information obtained from family, friends and online media platforms. This limited and unstructured access to information led them to hold overly optimistic beliefs about the success rate of ART. However, when treatment failed, they experienced a significant psychological gap and fell into deep self-doubt and distress. *N6: “I had some basic knowledge beforehand, but what I knew about the technology only came from what I saw on my phone.” N12: “Mostly what I heard from people around me, I guess, and what I read on Xiaohongshu (Little Red Book). I think watching videos about different treatment stages—like the embryo implantation stage—and scrolling through the app too much made me feel anxious. Now I try to be more selective with the information I consume and only read scientific content from reliable sources, which has helped a bit.” N9: “I actually knew very little about ART before coming to the clinic, even though I have a medical background. I only had a general understanding of the overall process but no knowledge of the specific details. I only learned the specifics after the doctor explained them to me.”*

#### 3.1.4 Plight of the stigma of disease

Influenced by traditional cultural beliefs, some participating women experienced an internal sense of shame when undergoing ART treatment, as they feared others would find out about their treatment experience. *N7: “We are an ethnic minority group, and our values are quite traditional. Even though some people around us have had IVF, it's still a very private matter—otherwise, people will judge us differently.” N13: “I don't think there is any shame in having IVF treatment, but I still don't want too many of my friends to know about it.” N15: “I still feel I can't let other people know that I'm unable to conceive naturally.”* With societal development, an increasingly open and tolerant social climate, and the gradual popularization of ART, the sense of shame associated with infertility has diminished among some women. *N11: “Maybe it's related to the changing social climate these days. It might have been shameful in the past, but now more and more people are having IVF, and this kind of thing seems to be becoming normalized.” N9: “Personally, I don't think it's a big deal because infertility is becoming more common these days. This kind of thing feels normal, and after all, many single mothers also opt for IVF treatment.”*

### 3.2 Theme 2: objective stress dilemmas

#### 3.2.1 Dilemmas in the social support environment

The lengthy ART treatment cycle requires participants to travel to and from the hospital frequently for injections, examinations and other procedures, which creates prominent conflicts with their work schedules. Notably, 10 out of 15 participants (67%) were unemployed at enrollment; this high unemployment rate shapes the observed work-related stress: employed respondents reported concrete conflicts between regular working attendance and recurrent hospital appointments, while unemployed participants escaped schedule conflicts yet confronted intensified long-term financial pressure stemming from lost household income. Many participants have to sacrifice their jobs to ensure the smooth progress of treatment, and some even face the risk of unemployment and compromised workplace status. *N9: “I think the biggest sacrifice I made was my job. I was actually earning a good salary, but IVF treatment is a very long process, which meant I couldn't keep the job, so I eventually quit.” N11: “Taking time off work constantly wasn't feasible, so I had to quit to focus on my treatment.” N7: “My health isn't very good and I have anemia. If I had to juggle frequent hospital visits with work, my body wouldn't be able to cope.” N3: “It used to be easy to take time off work, but now it's very difficult. I have to make up excuses every time I need to take time off for hospital visits.”*

Family support is crucial during ART treatment, yet some participants reported a lack of such support, particularly low spousal involvement. This leaves women to endure the physical and psychological stress of treatment alone, and undermines the intimacy of their marital relationship. *N14: “It seems like the men aren't really involved in the entire treatment process. I'm the one who has to do everything, so my spouse doesn't even understand the pain and stress I'm going through.” N12: “I still feel my husband's involvement is quite minimal—he only comes for the occasional check-up. It feels like this isn't something that concerns him much, and he can't take my place for the injections or tests, so it's definitely taking a toll on our relationship.”*

#### 3.2.2 Dilemmas of treatment-related costs

The high cost of ART treatment places a significant financial burden on participants and their families. The protracted nature of treatment requires ongoing financial investment, and if treatment fails, participants have to spend a significant amount of money to start a new cycle of treatment. This poses a tremendous financial strain on the average family. *N11: “Because it's so expensive, it's actually quite stressful.” N3: “I think it's too costly, and while it's true that Medicare currently reimburses, it's only a few of the things.” N15: “Since we are not in a good financial situation and the doctor informed that we need to continue if we are not successful the first time, I will just try my best to try once, I just can't afford it.”*

In addition to the financial costs, the time costs are also a heavy burden for participants to bear. Frequent hospital visits take up a great deal of participants' time, disrupt their daily routines, and create significant inconvenience in their lives. *N9: “There were so many runs on this day that it made me feel like a bit of a waste of time.” N14: “It takes an entire day to get to the hospital, and staying in a hotel is a bit inconvenient and a huge waste of time.” N7: “In the morning I take a taxi over to the hospital, I need to get up very early, I have to get up at 5:30 a.m. Not only does the quality of my sleep deteriorate, but the whole process is too time consuming for me to do alone.”*

### 3.3 Theme 3: dilemmas in stress coping styles

#### 3.3.1 Dilemmas of self-regulation

Faced with the stress of the ART treatment process, most participants attempt to alleviate their negative emotions through self-regulation, yet they generally lack scientific and effective self-regulation strategies to achieve the desired outcomes. *N9: “There's no specific way for me to cope with it except to tell myself not to think about the stress.” N12: “The first time an embryo transfer fails is especially tough, and it left me feeling somewhat resistant later on. The treatment process is so long and full of uncertainty—you never know what the final outcome will be, so I just have to talk myself out of overthinking it.” N7: “I just take my time to process my feelings. Ultimately, it means I fall apart emotionally first, then I try to work things out.”*

#### 3.3.2 Dilemmas of inadequate external support

Some participants have tried seeking professional psychological counseling, yet the results have been unsatisfactory. Such counseling fails to address the core emotional struggles of participants effectively, and the advice given is irrelevant and impractical, making it hard to provide truly effective psychological support. *N1: “I've been to counseling before, but the counselor's advice just felt like things I already do on my own anyway—it didn't help much.”*

Communicating with family members is an important way to relieve stress, yet miscommunication places an additional burden on participants. While family members showed concern, their well-meaning concern is often counterproductive because they lack a thorough understanding of ART treatment. *N15: “Sometimes when I'm in a bad mood and want to talk to my family, they really do care about me, but they don't understand what I'm going through. They keep asking for updates on the treatment, and hearing those questions only adds to my stress.”*

## 4 Discussion

### 4.1 An in-depth analysis of the subjective stress dilemma

#### 4.1.1 Exploring the causes of negative emotion

The lengthy cycle of assisted reproduction treatment and the high degree of uncertainty about the outcome are key factors in triggering negative emotions. Studies have shown (11) that uncertainty about the outcome of treatment is a major trigger for negative emotions, and women often experience emotional reactions such as anxiety and depression when faced with failure. During the treatment process, women need to go through a number of complicated stages, such as ovulation promotion, egg retrieval and embryo transfer, etc., each of which is accompanied by physical discomfort and psychological suffering. Especially in the post-ovulation and post-embryo transfer stages, a woman's expectation for the success of the treatment reaches its peak, and once the result is not as expected, she is very prone to anxiety and worry.

From the perspective of mental toughness theory (12), an individual's psychological resilience determines how they cope with ongoing stress and setbacks. In this sample, participants not only bore the stress brought by repeated treatment attempts and uncertain outcomes, but also faced persistent fertility expectations from elder family members under traditional cultural norms (13). For women with low psychological resilience, these two types of stress overlapped and amplified each other, resulting in more intense anxiety and distress after treatment failure. This explains why participants such as N12 and N13 expressed profound grief after setbacks: their limited resilience could not buffer the combined pressure from treatment itself and family expectations. It also reflects that in the local cultural context, fertility-related demands become an additional burden that tests women's stress tolerance during ART.

#### 4.1.2 Multidimensional effects of body image change

During the process of assisted reproduction treatment, a series of changes will occur in a woman's body, such as obesity and hardening at the injection site, etc. These bodily changes will have a profound impact on a woman's self-perception and psychological state (14). Obesity not only affects women's outward appearance, but can also trigger dissatisfaction and low self-esteem about their bodies (15). Hard knots at the injection site, on the other hand, make women fearful and anxious about the treatment, worrying that their bodies will suffer long-term damage as a result (16). Socio-cultural aesthetic standards of body image play a contributing role, with the dominant aesthetic promoting a slimmer figure making women more sensitive and uncomfortable with treatment-induced obesity (17).

Body image theory (18) states that people's evaluation of their own bodies is shaped by both personal feelings and the surrounding social environment. In Chinese collectivist culture, individuals pay great attention to external evaluations from relatives and friends, which is distinctly different from the self-oriented body perception in Western individualistic societies (19). As seen in participant N11's experience, weight gain caused by treatment not only led to personal dissatisfaction, but also triggered worry about being judged by others. This social pressure further deepened her inferiority and anxiety, and in turn made her more sensitive to physical changes, forming a vicious cycle. The body image changes observed in this study are not merely physical problems; they are transformed into persistent psychological stress under the combined effect of social aesthetics and interpersonal concerns, which fully aligns with the core connotation of body image theory.

#### 4.1.3 Sources and effects of cognitive biases

Most women have limited knowledge of ART prior to treatment, relying mainly on friends, relatives and online media for relevant information. Such fragmented and unregulated information is often one-sided and inaccurate, which gives rise to overly optimistic expectations. When treatment fails, the huge psychological gap leads to self-doubt and intensified stress. Cognitive bias in this study also interfered with patients' communication with medical staff and their treatment decisions.

According to information asymmetry theory (20), unequal access to professional information will easily make individuals misled by informal content. Unlike Western patients who mainly obtain ART knowledge from official medical channels, participants in this study primarily learned about treatment through social platforms and word-of-mouth communication, resulting in prominent information asymmetry This explains why most participants held unrealistic high hopes for treatment success before enrollment. In addition, participants with lower educational attainment (e.g., N7, N11) lacked the ability to distinguish true and false online information, so their cognitive biases were more obvious after receiving fragmented content (21). Combined with the interview accounts of N6 and N12, it can be concluded that the local information acquisition mode directly shapes patients' cognitive deviations, which is a typical manifestation of information asymmetry in this clinical scenario (22).

#### 4.1.4 Socio-cultural roots of stigma

In many socio-cultural contexts, fertility is regarded as an important responsibility of women (22). Although ART brings hope to infertile patients, traditional concepts still make this group face invisible stigma. Some participants chose to hide their treatment experience for fear of being gossiped by others, and this internal shame became an extra psychological burden. Such a sense of stigma is more prominent among ethnic minority groups with more conservative ideas.

Socio-cultural theory illustrates that traditional values and social cognition can profoundly affect individuals' behaviors and inner feelings (22). In the cultural atmosphere where “having offspring is essential”, infertility is easily regarded as a personal regret, and ART is also treated as a private and embarrassing matter by part of the public. Even though social openness and the popularization of ART have gradually weakened such prejudice, long-established cultural inertia still makes the sense of stigma difficult to eliminate in a short time. This accounts for why some participants still chose to conceal their treatment status. Relevant studies have proven that public science popularization can optimize social cognition and relieve stigma stress (23), which also provides a feasible way to improve the current situation in this region.

### 4.2 A comprehensive analysis of the objective pressure dilemma

#### 4.2.1 A multifactorial exploration of the lack of support

Work conflict is an important objective pressure faced by women during assisted reproduction treatment. The long treatment cycle and the need to travel frequently to and from the hospital create a serious conflict with normal working hours, making it difficult for women to balance work and treatment (15). Some women have to reduce their working hours or even give up their jobs in order to ensure the effectiveness of their treatment, which adversely affects their career development and financial income. It should be noted that the sample's high unemployment proportion (67%) limits universal interpretation of work-treatment conflict findings: the notable trend of participants resigning from work observed in this cohort cannot be directly generalized to populations with higher employment rates, as fully employed women are protected by workplace leave policies and encounter different types of scheduling strain rather than complete job loss.

Lack of family support, especially low spousal involvement, is also a major challenge for women in the treatment process (24). Social support theory holds that effective support from work and family can buffer various stresses and improve individuals' ability to cope with difficulties (18). In this study, frequent hospital visits forced many participants to quit their jobs, depriving them of stable occupational support; meanwhile, low spousal participation meant women could not obtain the most intimate emotional comfort. As reflected in the narratives of N12 and N14, when the core support system failed to function, participants had to bear all the physical and mental pressure alone, which significantly increased their anxiety. This is consistent with existing research conclusions: adequate work and family support can effectively reduce treatment-related anxiety and improve treatment adherence (25).

#### 4.2.2 Financial and time burdens of treatment

The high cost of assisted reproduction treatment places a heavy financial burden on many families. From the cost of medication to the cost of surgery to the cost of follow-up examinations and care, each item is a significant expense. For families with limited financial resources, it may take everything they have to complete a treatment cycle, and if the treatment fails, the financial pressure to continue the treatment will be even more difficult to bear (26, 27). This economic pressure not only affects the continuity and success of women's treatment, but may also have a negative impact on the family's quality of life and social stability (26). The limited coverage of assisted reproduction treatment under the current health insurance policy is unable to effectively alleviate the financial burden on families, which also reflects the inadequacy of society's attention and support for this special medical need (22).

Economic stress theory points out that excessive financial pressure will directly impair individuals' mental well-being and life satisfaction (28). For the participants in this study, repeated treatment cycles meant continuous capital investment. When medical expenses occupied a large proportion of household income, patients had to worry about both treatment outcomes and family livelihood at the same time. As mentioned by N3 and N15, the high cost and limited medical reimbursement made them hesitate to receive further treatment after failure. This fully verifies the core viewpoint of economic stress theory: the heavier the family's economic burden brought by ART, the stronger the patients' psychological stress during treatment (24).

### 4.3 Assessment and reflection on the dilemma of stress coping styles

#### 4.3.1 Manifestations and causes of self-regulation dilemmas

In the face of the various stresses associated with the process of assisted reproduction treatment, most women try to alleviate their emotions through self-regulation. However, they generally report that they can only cope with the stress through simple self-soothing or temporary avoidance, which often only have a short-lived effect and cannot solve the problem at its root. Meanwhile, during the treatment process, the medical team mainly focuses on women's physical condition and treatment effect, and pays relatively little attention and support to the psychological aspect, which makes women lack effective coping strategies and guidance when facing psychological stress (29). Emotion regulation theory (30) states that individuals need to use effective regulation strategies, such as cognitive reappraisal and problem solving, to better cope with stress when facing negative emotions. However, this study found that most women mainly used emotion-focused regulation strategies, such as self-soothing and emotional venting, but less problem-focused regulation strategies during assisted reproduction therapy. This suggests that their ability in emotion regulation needs to be improved, while more psychological care needs to be provided by healthcare professionals.

#### 4.3.2 Difficulties in solving the lack of outside help

Psychological counselling in the field of assisted reproduction therapy is not yet well developed, and the existing system of psychological counselling services suffers from a lack of professionalism and a lack of relevance. Some psychological counselors lack in-depth knowledge of the specifics of assisted reproduction therapy and the psychological characteristics of women, and the counseling advice they provide fails to meet the actual needs of women, resulting in unsatisfactory psychological counseling results.

The causes of family communication barriers are complex and varied, including generational gaps, information asymmetry, and psychological stress on family members. Family members' lack of understanding of assisted reproduction treatment and their neglect of women's stress make it difficult for women to seek family support. Therefore, there is a need to strengthen the construction of professional psychological counseling teams to improve the quality and relevance of psychological counseling, as well as to carry out educational activities for family members to enhance their understanding of and ability to support assisted reproduction treatment (31).

## 5 Conclusions and limitations

This study found that the stressful dilemmas faced by women in the process of assisted reproductive technology (ART) treatment are characterized by multidimensionality and complexity, such as the special manifestation of disease stigma in a specific cultural context and the impact of cognitive bias on treatment decision-making, etc. These findings provide a useful supplement and expansion of existing theories, and offer a new perspective for a more comprehensive understanding of women's psychological experience in assisted reproduction treatment.

At the same time, this study provides important insights for optimizing clinical practice in the process of assisted reproductive technology treatment. First, in terms of psychological care, medical teams should strengthen their attention to women's psychological status, identify and intervene in negative emotions in a timely manner, and provide personalized psychological support and guidance. Secondly, patient education needs to be further strengthened, so that diverse educational activities can be carried out to improve women's correct knowledge of assisted reproductive technology and reduce the psychological pressure caused by cognitive bias. At the same time, it is also necessary to strengthen the construction of a family support system, encourage men to actively participate in the treatment process, and carry out education and support programs for family members to improve family support for women's treatment.

The present research was limited to a single tertiary hospital in Chongqing; hence, the findings cannot be generalized to infertile women across diverse regions, ethnic groups and socioeconomic strata nationwide. Multi-centre sampling covering different provinces, urban and rural areas is required in future research to improve sample representativeness. Moreover, the sample features a notably high unemployment rate (67%), which introduces sampling bias; the overrepresentation of unemployed participants amplifies the prevalence of financial burden-related dilemmas and restricts the transferability of work-stress findings to employed-dominant female populations. The study design failed to adequately consider the dynamic impact of the time factor on women's stress dilemma. Future multi-center longitudinal studies are recommended to track dynamic psychological changes among diverse groups. The single-site sampling design limits the transferability of current findings to infertile women from different regions, socioeconomic and ethnic backgrounds nationwide.

## Data Availability

The raw data supporting the conclusions of this article will be made available by the authors, without undue reservation.

## References

[1] Hu L Bu Z Huang G Sun H Deng C Sun Y. Assisted reproductive technology in China: results generated from data reporting system by CSRM from 2013 to 2016. Front Endocrinol (Lausanne). (2020) 11:458. 10.3389/fendo.2020.00458 33042000 PMC7527788

[2] Tang Z. Confucianism, Chinese culture, and reproductive behavior. Popul Environ. (1995) 16(3):269–84. 10.1007/BF02331921

[3] Jing X Gu W Zhang L Miao R Xu X Wang M. Coping strategies mediate the association between stigma and fertility quality of life in infertile women undergoing *in vitro* fertilization-embryo transfer. BMC women's Health. (2021) 21(1):386. 10.1186/s12905-021-01525-9 34727911 PMC8561985

[4] Wu L Sun L Wang J Zhang X Huang Y Lu Y. Psychological distress among women undergoing *in vitro* fertilization-embryo transfer: a cross-sectional and longitudinal network analysis. Front Psychol. (2022) 13:1095365. 10.3389/fpsyg.2022.1095365 36687877 PMC9849569

[5] Sletteboe A. Dilemma: a concept analysis. J Adv Nurs. (1997) 26(3):449–54. 10.1046/j.1365-2648.1997.t01-1-00999.x 9378862

[6] Stanhiser J Steiner AZ. Psychosocial aspects of fertility and assisted reproductive technology. Obstet Gynecol Clin North Am. (2018) 45(3):563–74. 10.1016/j.ogc.2018.04.006 30092929

[7] Gdańska P Drozdowicz-Jastrzębska E Grzechocińska B Radziwon-Zaleska M Węgrzyn P Wielgoś M. Anxiety and depression in women undergoing infertility treatment. Ginekol Pol. (2017) 88(2):109–12. 10.5603/GP.a2017.0019 28326521

[8] Ozturk A Aba YA Sik BA. The relationship between stigma, perceived social support and depression in infertile Turkish women undergoing *in vitro* fertilization-embryo transfer. Arch Psychiatr Nurs. (2021) 35(5):434–40. 10.1016/j.apnu.2021.05.009 34561056

[9] Santos G Gottschang SZ. Rethinking reproductive technologies and modernities in time and space. Technol Cult. (2020) 61(2):549–58. 10.1353/tech.2020.0052 33416777

[10] Englander M Morley J. Phenomenological psychology and qualitative research. Phenomenol Cogn Sci. (2023) 22(1):25–53. 10.1007/s11097-021-09781-8 34744533 PMC8556824

[11] Purewal S Chapman SCE Van den Akker OBA. Depression and state anxiety scores during assisted reproductive treatment are associated with outcome: a meta-analysis. Reprod Biomed Online. (2018) 36(6):646–57. 10.1016/j.rbmo.2018.03.010 29622404

[12] Schwerdtfeger KL Shreffler KM. Trauma of pregnancy loss and infertility for mothers and involuntarily childless women in the contemporary United States. J Loss Trauma. (2009) 14(3):211–27. 10.1080/15325020802537468 21686042 PMC3113688

[13] Mijalevich-Soker E Meir R Taubman—Ben-Ari O. The long path to motherhood: personal growth and well-being during medically assisted reproduction. Behav Sci. (2025) 15(12):1694. 10.3390/bs15121694 41464037 PMC12729309

[14] Zhang X Yang L Wang W Yang L. Psychological distress, emotion regulation, neuroticism, and sexual relationship on patients with temporary ejaculation failure *in vitro* fertilization-embryo transfer treatment. Front Psychol. (2022) 13:1090244. 10.3389/fpsyg.2022.1090244 36687954 PMC9853009

[15] Lykeridou K Gourounti K Sarantaki A Loutradis D Vaslamatzis G Deltsidou A. Occupational social class, coping responses and infertility-related stress of women undergoing infertility treatment. J Clin Nurs. (2011) 20(13-14):1971–80. 10.1111/j.1365-2702.2011.03696.x 21564361

[16] Zhan FZ Yang HL Sun XL Zhu YC Meng FF. Depression and its associated factors among infertile patients undergoing assisted reproductive technology. Chin J Hum Sex. (2023) 32(6):143–7. 10.3969/j.issn.1672-1993.2023.06.035

[17] Ye S. The impact of enhanced psychological intervention on the psychological well-being and family functioning of infertile patients undergoing assisted reproductive technology. Contemp Chin Med. (2021) 28(13):4. 10.3969/j.issn.1006-7256.2022.10.052

[18] Yu CP Li WL Deng MF. Hope and anxiety: the study of female embodied experience with ARTs. Chin J Sociol. (2019) 39(4):84–115. Available online at: https://www.society.shu.edu.cn/CN/

[19] Cash TF. Cognitive-behavioral perspectives on body image. In: Cash TF, editor. Encyclopedia of Body Image and Human Appearance: Vol.1. San Diego: Academic Press (2012). p. 334–42. 10.1016/B978-0-12-384925-0.00054-7

[20] Gupta A Lu E Thayer Z. The influence of assisted reproductive technologies-related stressors and social support on perceived stress and depression. BMC women's Health. (2024) 24(1):431. 10.1186/s12905-024-03262-1 39068405 PMC11282751

[21] Goisis A Fallesen P Seiz M Salazar L Eremenko T Cozzani M. Educational gradients in the prevalence of medically assisted reproduction births in a comparative perspective. Fertil Steril. (2024) 122(4):648–57. 10.1016/j.fertnstert.2024.05.149 38768747 PMC11452323

[22] Johnson-Ekeleba AC Sefogah PE Swarray-Deen A Mumuni K. Awareness and acceptability of assisted reproductive technology among non-medical tertiary students in a low-resource setting. Reprod Biol Endocrinol. (2024) 22(1):131. 10.1186/s12958-024-01292-w 39468518 PMC11514781

[23] Wang Q Jia D Gao Y Zhou M Zhao X Qin R. Relationship between stigma and infertility-related stress among couples undergoing AID: the mediating role of communication patterns. Stress Health. (2024) 40(5):e3412. 10.1002/smi.3412 38651677

[24] Donarelli Z Lo Coco G Gullo S Marino A Volpes A Allegra A. Are attachment dimensions associated with infertility-related stress in couples undergoing their first IVF treatment? A study on the individual and cross-partner effect. Hum Reprod. (2012) 27(11):3215–25. 10.1093/humrep/des307 22926837

[25] Ma XL. Analysis of Key Factors Influencing Anxiety and Depression in Patients Undergoing Assisted Reproductive Treatment. Henan: Henan University (2023).

[26] Ethics Committee of the American Society for Reproductive Medicine. Financial ‘‘risk-sharing’’ or refund programs in assisted reproduction: an ethics committee opinion. Fertil Steril. (2024) 121(5):783–6. 10.1016/j.fertnstert.2023.12.032 38276940

[27] Leroux ML Pestieau P Ponthiere G. The optimal design of assisted reproductive technologies policies. Health Econ. (2024) 33(7):1454–79. 10.1002/hec.4822 38475875

[28] Meyers AJ. Mental health screening and psychological support should be the standard of care in fertility clinics. J Assist Reprod Genet. (2025) 42:2285–91. 10.1007/s10815-025-03543-0 40528070 PMC12356772

[29] Jiang L Zeng T Wu M Yang L Zhao M Yuan M. Infertility psychological distress in women undergoing assisted reproductive treatment: a grounded theory study. J Clin Nurs. (2024) 33(9):3642–58. 10.1111/jocn.17195 38716811

[30] Gross JJ. Emotion regulation: affective, cognitive, and social consequences. Psychophysiology. (2002) 39(3):281–91. 10.1017/S0048577201393198 12212647

[31] Zhai QL Wang CY Liu F Mei J Yuan S. The effect of family-participatory nursing on the sense of stigma among patients undergoing assisted reproductive technology. Nurs Integr Tradit Chin West Med. (2023) 9(4):165–7. 10.11997/nitcwm.202304050

